# Impaired psychological recovery in the elderly after the Niigata-Chuetsu Earthquake in Japan:a population-based study

**DOI:** 10.1186/1471-2458-6-230

**Published:** 2006-09-14

**Authors:** Shin-ichi Toyabe, Toshiki Shioiri, Hideki Kuwabara, Taroh Endoh, Naohito Tanabe, Toshiyuki Someya, Kouhei Akazawa

**Affiliations:** 1 Department of Medical Informatics, Niigata University Medical and Dental Hospital, Asahimachi-Dori 1, Niigata 951–8520, Japan; 2 Department of Psychiatry, Niigata University Graduate School of Medical and Dental Sciences, Asahimachi-Dori 1, Niigata 951–8510, Japan; 3 Department of Health Promotion, Niigata University Graduate School of Medical and Dental Sciences, Asahimachi-Dori, Niigata 951–8510, Japan

## Abstract

**Background:**

An earthquake measuring 6.8 on the Richter scale struck the Niigata-Chuetsu region of Japan at 5.56 P.M. on the 23rd of October, 2004. The earthquake was followed by sustained occurrence of numerous aftershocks, which delayed reconstruction of community lifelines. Even one year after the earthquake, 9,160 people were living in temporary housing. Such a devastating earthquake and life after the earthquake in an unfamiliar environment should cause psychological distress, especially among the elderly.

**Methods:**

Psychological distress was measured using the 12-item General Health Questionnaire (GHQ-12) in 2,083 subjects (69% response rate) who were living in transient housing five months after the earthquake. GHQ-12 was scored using the original method, Likert scoring and corrected method. The subjects were asked to assess their psychological status before the earthquake, their psychological status at the most stressful time after the earthquake and their psychological status at five months after the earthquake. Exploratory and confirmatory factor analysis was used to reveal the factor structure of GHQ12. Multiple regression analysis was performed to analyze the relationship between various background factors and GHQ-12 score and its subscale.

**Results:**

GHQ-12 scores were significantly elevated at the most stressful time and they were significantly high even at five months after the earthquake. Factor analysis revealed that a model consisting of two factors (social dysfunction and dysphoria) using corrected GHQ scoring showed a high level of goodness-of-fit. Multiple regression analysis revealed that age of subjects affected GHQ-12 scores. GHQ-12 score as well as its factor 'social dysfunction' scale were increased with increasing age of subjects at five months after the earthquake.

**Conclusion:**

Impaired psychological recovery was observed even at five months after the Niigata-Chuetsu Earthquake in the elderly. The elderly were more affected by matters relating to coping with daily problems.

## Background

An earthquake measuring 6.8 on the Richter scale struck the Niigata-Chuetsu region of Japan at 5.56 P.M. on the 23rd of October, 2004. The earthquake was followed by sustained occurrence of numerous aftershocks, one of which measuring 5.0 occurred even on 28th of December, 2004. The earthquake and the following aftershocks left more than 4,500 injured and 120,000 houses completely or partially destroyed. About 100,000 people were displaced from their homes, and some of them moved into temporary housing. Because of the sustained occurrence of aftershocks and delayed reconstruction of community lifelines, 9,160 people who lost their houses were still living in temporary housing even in November 2005, one year after the earthquake. The impact of the devastating earthquake and the following life in an unfamiliar environment should cause psychological distress for people affected by the earthquake [1-5]. Therefore, there was a need to identify the group at high risk for psychological distress after the earthquake [2]. We measured psychological distress using the 12-item General Health Questionnaire (GHQ-12), which is a widely used screening instrument for mental disorders [6,7], among people who were affected by the Niigata-Chuetsu Earthquake and had been living in temporary housing.

Previous studies have shown that the elderly are at high risk for psychological distress as a result of a large disaster [2,8]. However, it is not known what aspects of psychological distress are more affected in the elderly than in younger people. We studied the change in GHQ-12 score over time after the earthquake and analyzed the factor structure of the score [6,9-15]. Our results showed that the elderly had greater impairment in recovery from psychological distress, even five months after the earthquake, than did younger subjects. Factor analysis suggested that 'social functioning' was more impaired in the elderly than in younger subjects.

## Methods

Five months after the earthquake, 3,026 subjects who lost houses at the earthquake and had continued to live in temporary housing were asked to reply to questionnaire surveys prepared to measure their psychological distress. A total of 2,083 subjects replied to the questionnaire, a response rate of 69%. Characteristics of the study subjects are outlined in Table 1. Psychological distress was measured using the Japanese version of the 12-item General Health Questionnaire (GHQ-12) [6,7]. To evaluate the changes in psychological distress over time, the subjects were asked to assess their mental state before the earthquake (pre-earthquake), at the time when the subject felt the most stressful after the earthquake (post-earthquake) and at the time of replying to the questionnaire five months after the earthquake (now). The GHQ-12 was scored by the original (0-0-1-1) method (GHQ), corrected scoring (0-1-1-1) by Goodchild (C-GHQ) [16] and Likert (0-1-2-3) scoring [15].

To study factor structure of GHQ-12, exploratory factor analysis was performed [3,6,9-15,17-27]. We used the Promax rotation method because there might be inter-factor correlations [15, 26, 28]. Factors with eigenvalue more than 1.0 were accepted. Internal consistency of a series of items belonging to each factor was evaluated using Cronbach's alpha score [29]. If Cronbach's alpha score of a factor was more than 0.7, we considered internal consistency of the factor to be satisfactory. In that case, we calculated lower scale points for each factor by averaging scales of all items belonging to the factor. Confirmatory factor analysis was conducted using AMOS 5 (SPSS Japan Inc., Tokyo Japan) to test the fits of models derived from the results of exploratory factor analysis [30]. Since AMOS does not provide tetrachoric correlations, we used binary data as continuous data in spite of problems over handling binary data in factor analysis [31, 32]. Goodness-of-fit of the models was tested by using F0 (estimated population discrepancy), root mean square error of approximation (RMSEA) and ECVI (expected cross-validation index) [33].

Multiple regression analysis was conducted to evaluate the impact of subjects' background on the GHQ-12 scores. Independent variables used in the analysis are shown in Table 1. Categorical variables and ordered variables were converted to dummy variables. Analysis of variance (ANOVA) with Scheffe post hoc tests was used to evaluate differences over time in the GHQ-12 scores as well as lower scale points of the identified factors. In all tests, a p value less than 0.05 was considered as statistically significant. Analysis other than confirmatory factor analysis was performed using SPSS 14.0J.

This study was approved by the Ethics Committee of Niigata Graduate School of Medical and Dental Sciences. Informed written consent was obtained from all subjects.

## Results

**Figure 1:**
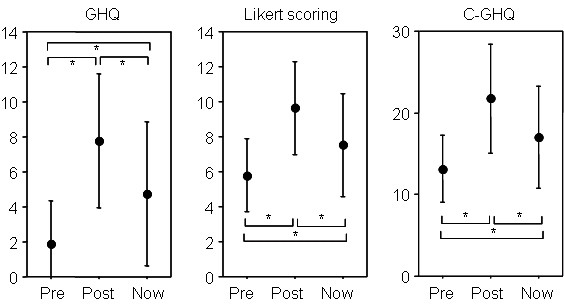
**Time course of GHQ-12 scored by three different methods**. GHQ-12 scores were assessed five months after the earthquake, and the subjects were required to assess mental state before the earthquake (Pre), mental state at the most stressful time after the earthquake (Post) and mental state at the time of assessment (Now). Mean and standard deviation values are shown. Differences between scores at the three points of time were analyzed by ANOVA with Scheffe post hoc analysis. A p-value less than 0.01 is indicated by an asterisk (*).

GHQ-12 scored by the three scoring methods was significantly elevated when the subjects felt the most stressful compared to the scores before the earthquake. Although the scores had decreased five months after the earthquake, they were still higher than the scores before the earthquake (Fig. 1).

Exploratory factor analyses revealed that two factors were derived from GHQ-12 scores of three points of time (Table 2). Each of the two factors consisted of the same items regardless of the scoring method and timing of assessment. Factor I consisted of items 1, 3, 4, 7 and 8, and factor II consisted of items 2, 5, 6, 9, 10 and 11. This two-factor structure was the same as the two-factor model developed by Doi [6]. We defined factor I as 'social dysfunction' related to the ability to cope with everyday problems and factor II as 'dysphoria' related to anxiety and depression according to previous studies [6,9-15]. Item 12 ('feeling reasonably happy') did not belong to either factor I or factor II. There were no or only weak correlations between the factors when C-GHQ scoring was used. On the other hand, comparatively strong correlations were found between the factors when the other scoring methods were used.

Confirmatory factor analysis using the two-factor model showed favorable model fitting in terms of three measures for goodness-of-fit regardless of the difference in the scoring methods (Table 3). Although there were no significant differences (α = 0.1) in the three fitting measures between the three scoring methods, the C-GHQ method generally produced the most optimal fitting measures compared with the original GHQ and Likert scoring, except RMSEA evaluated at five months after the earthquake. Therefore, we used the C-GHQ score in the subsequent analyses. Cronbach's alpha scores for the two factors ranged from 0.85 to 0.91 in C-GHQ scores at the three points of time. Therefore, we considered that the internal consistency of the two factors was sufficient at all three points of time. We calculated the lower scale points for each factor by averaging the scores of all items belonging to the factor.

**Figure 2:**
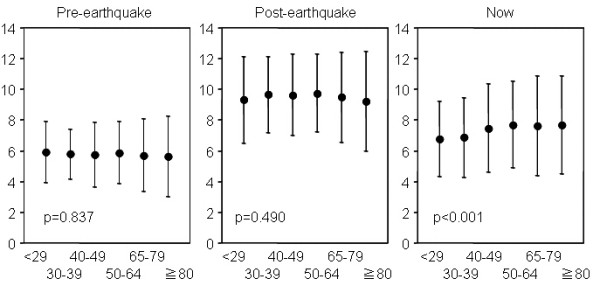
**Difference in C-GHQ scores by age**. C-GHQ scores before the earthquake (pre-earthquake), at the most stressful time after the earthquake (post-earthquake) and five months after the earthquake (now) are shown as mean and standard deviation values. In each graph, plots from left to right correspond to the six age groups shown in Table 1. The effect of age of subjects on the GHQ-12 scores was analyzed by ANOVA. The p value in each test is shown in the plot.

**Figure 3:**
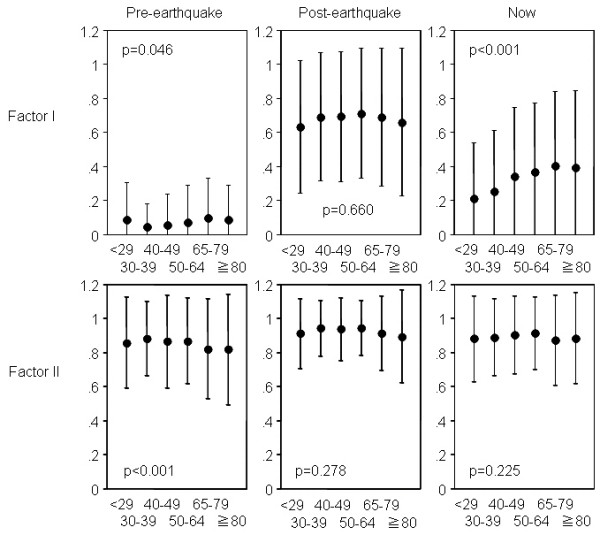
**Differences in lower scale points of each factor by age group of subjects**. The relationships between subjects' age and factor I or factor II points of pre-earthquake, post earthquake and now are shown as mean and standard deviation values. In each graph, plots from left to right correspond to the six groups of age shown in Table 1. Trends of GHQ-12 scores with increasing age of subjects were analyzed by the ANOVA. The p value in each test is shown in the plot.

Next, we analyzed the factor that affects GHQ-12 scores at the three points of time using multiple regression analysis with dummy variables. Table 4 shows regression coefficients of each dependent variable that significantly affect GHQ-12 score. The results of the analysis showed that GHQ-12 scores were associated with various factors, including age of subjects. The results of ANOVA showed that there was a tendency for the GHQ-12 score at five months after the earthquake to increase with increasing age of subjects (Fig. 2). On the other hand, there were no differences between age groups in GHQ-12 score before and after the earthquake. We then analyzed which factor was more affected by age of subjects, considering the results of factor analysis. As shown in Table 4, the results of multiple regression analysis showed that the lower scale point at factor I five months after the earthquake was significantly affected by age of subjects, whereas that of factor II was not (Fig. 3). The lower scale points of the two factors before the earthquake showed different change with increasing age of subjects (Fig.3). In the case of GHQ-12 before the earthquake, the lower scale point of factor I increased with increasing age of subjects, but that of factor II decreased.

## Discussion

In the present study, we found that the elderly had greater impairment in recovery from psychological distress than did younger subjects after the Niigata-Chuetsu Earthquake. The level of psychological morbidity assessed using GHQ-12 was lower than that before the earthquake even at five months after the earthquake. At that point of time, the elderly were more affected by matters relating to coping with daily problems, as shown by confirmatory factor analysis of GHQ-12.

Previous studies have suggested that there are some predictors of psychological morbidity after an earthquake [2,4]. The elderly [2,8], females [34] and subjects exposed to disruption are at risk for development of psychological distress [2]. Exposure to disruption is estimated by location at the time of the earthquake. Our results of multiple regression analysis suggest that various factors, including the abovementioned risk factors, affect psychological outcome at five months after the earthquake. We found that aging was a risk factor that impaired recovery from psychological distress at five months after the Niigata-Chuetsu Earthquake. The elderly were more affected by matters relating to coping with daily problems than were younger subjects, as shown by the results of factor analysis.

We found that the two-factor model using C-GHQ scoring was best fitted to scores of the three points of time. Moreover, the impairment of recovery from psychological distress in the elderly was obvious in the factor 'social dysfunction' but was not obvious in the factor 'dysphoria'. GHQ-12 is widely used as a uni-dimensional instrument [9,11], but two or three factors in GHQ-12 have been identified in previous studies [9,12,14]. The most common factors that have been identified are a factor for anxiety and depression and a factor for social dysfunction. In general, the factor structure of GHQ-12 has provided quite different results in terms of scoring methods, clinical groups and different cultures [12]. However, the factor structure in the present study was quite stable regardless of the difference in scoring methods. Some researchers have demonstrated that their factor model showed a good fit for C-GHQ scoring compared to that for scoring by other methods [9,16]. Our results confirm the results of these studies. Moreover, the weak correlation between the factors suggests that GHQ-12 is a multi-dimensional instrument when applied to the subjects who suffered in the Niigata-Chuetsu Earthquake. Using the factors separately offers a practical advantage in identifying psychological problems in the elderly.

There were some limitations of our study. First, only data from subjects who were affected by the earthquake and continued to live in temporary housing were used for analysis. Data for subjects less affected or unaffected by the earthquake were not included in our study. Second, the pre-earthquake and post-earthquake GHQ-12 scores were assessed five months after the earthquake. The pre-earthquake and post-earthquake GHQ-12 scores might not be accurate because they were assessed while remembering past events. Self perceptions of well-being before the earthquake are likely to be affected by how individuals are currently feeling. That is why the elderly might underestimate matters relating to pre-earthquake factor II. For the same reason, some post-earthquake items were associated with pre-earthquake GHQ scores in multiple regression analysis. Many studies have suggested that aging influences memories [35, 36, 37]. Nevertheless, it is obvious that the elderly were more affected by matters related to factor I even five months after the earthquake and that special care is needed for the elderly who suffered in the earthquake in order to resolve these problems.

## Conclusion

Impaired psychological recovery was observed even at five months after the Niigata-Chuetsu Earthquake in the elderly. The elderly were more affected by matters relating to coping with daily problems.

## Competing interests

The author(s) declare that they have no competing interests.

## Authors' contributions

ST contributed to concept and design of the article and analysis of data. TS1, HK and TE contributed to acquisition of data. NT contributed to statistical analysis of data. TS2 and AK supervised all aspects of the study and revised the article. All authors read and approved the final manuscript.

## Pre-publication history

The pre-publication history for this paper can be accessed here:



## Figures and Tables

**Table 1:** Characteristics of the subjects (n = 2,083)

		N	%
Gender	Male	1,137	54.6
	Female	927	44.5
Age (years)	≤29	59	2.8
	30–39	184	8.8
	40–49	333	16.0
	50–64	802	38.5
	65–79	623	29.9
	≥80	66	3.2
Place of residence when the earthquake occurred			
	Nagaoka City	727	34.9
	Ojiya City	442	21.2
	Mitsuke City	171	8.2
	Tohkamachi City	248	11.9
	Kawaguchi Town	146	7.0
	Koshiji Town	108	5.2
	Yamakoshi Village	216	10.4
Employment	Farmer	236	11.3
	Executive of business firm	128	6.1
	Office worker	461	22.1
	Government official	54	2.6
	Part-time worker	167	8.0
	Housewife	303	14.5
	Student	5	0.2
	None	201	9.6
	Pensioner	295	14.2
Location when the earthquake occurred			
	At home	1,426	68.5
	At home of friends	30	1.4
	At office	132	6.3
	In Car	169	8.1
	In bus or train	8	0.4
	In public facilities	29	1.4
	In other buildings	71	3.4
	Outdoors	78	3.7
Person by their side when the earthquake occurred			
	With someone	317	15.2
	Alone	1,766	84.8
Family members living together			
	None	847	40.7
Income			
	Lost income after earthquake	21	1.0
Severity of house damage	Completely destroyed	356	17.1
	Almost completely destroyed	292	14.0
	Severely damaged	621	29.8
	Slightly damaged	787	37.8
	None	14	0.7
Timing of contact with lifeguards after the earthquake			
	≤1 hour	139	6.7
	1< ≤ 3 hours	263	12.6
	3< ≤ 6 hours	187	9.0
	6< ≤ 12 hours	204	9.8
	> 12 hours	998	47.9
	No contact	279	13.4
Injury caused by the earthquake	No injury	1,888	90.6
	Mild injury	191	9.2
	Injury requiring hospitalization	4	0.2
Sickness after the earthquake	No sickness	1,870	89.8
	Mild sickness	195	9.4
	Sickness requiring hospitalization	18	0.9

**Table 2:** Factor loadings

	GHQ-12 items	GHQ		Likert		C-GHQ	
		F1	F2	F1	F2	F1	F2
	1. Able to concentrate	0.68	-	0.66	-	0.66	-
	2. Lost much sleep	-	0.62	-	0.65	-	0.62
	3. Playing a useful part	0.69	-	0.71	-	0.70	-
	4. Capable of making decisions	0.77	-	0.81	-	0.75	-
	5. Under stress	-	0.71	-	0.76	-	0.76
	6. Could not overcome difficulties	-	0.69	-	0.71	-	0.75
Before earthquake	7. Enjoy normal activities	0.76	-	0.72	-	0.78	-
	8. Face up to problems	0.72	-	0.77	-	0.74	-
	9. Feeling unhappy and depressed	-	0.82	-	0.84	-	0.81
	10. Losing confidence	-	0.72	-	0.78	-	0.81
	11. Thinking of self as worthless	-	0.48	-	0.52	-	0.58
	12. Feeling reasonably happy	-	-	-	-	-	-
	Inter-factor correlation	0.43		0.43		0.13	
	1. Able to concentrate	0.76	-	0.73	-	0.76	-
	2. Lost much sleep	-	0.55	-	0.63	-	0.59
	3. Playing a useful part	0.73	-	0.79	-	0.80	-
	4. Capable of making decisions	0.77	-	0.81	-	0.81	-
	5. Under stress	-	0.58	-	0.78	-	0.79
	6. Could not overcome difficulties	-	0.66	-	0.73	-	0.82
Most stressful time	7. Enjoy normal activities	0.80	-	0.69	-	0.80	-
	8. Face up to problems	0.83	-	0.84	-	0.85	-
	9. Feeling unhappy and depressed	-	0.84	-	0.89	-	0.85
	10. Losing confidence	-	0.78	-	0.76	-	0.82
	11. Thinking of self as worthless	-	0.49	-	0.48	-	0.48
	12. Feeling reasonably happy	-	-	-	-	-	-
	Inter-factor correlation	0.74		0.71		0.40	
	1. Able to concentrate	0.77	-	0.73	-	0.77	-
	2. Lost much sleep	-	0.70	-	0.72	-	0.63
	3. Playing a useful part	0.82	-	0.79	-	0.82	-
	4. Capable of making decisions	0.83	-	0.81	-	0.84	-
	5. Under stress	-	0.78	-	0.82	-	0.76
	6. Could not overcome difficulties	-	0.66	-	0.77	-	0.80
Now	7. Enjoy normal activities	0.73	-	0.71	-	0.80	-
	8. Face up to problems	0.78	-	0.77	-	0.83	-
	9. Feeling unhappy and depressed	-	0.85	-	0.91	-	0.86
	10. Losing confidence	-	0.72	-	0.85	-	0.86
	11. Thinking of self as worthless	-	0.40	-	0.47	-	0.55
	12. Feeling reasonably happy	-	-	-	-	-	-
	Inter-factor correlation	0.73		0.70		0.30	

Factor analysis was conducted for GHQ-12 scores at three points of time. GHQ-12 was scored by using the three different methods.

**Table 3:** Fit measures of models derived from factor analysis

	scoring methods	F0	RMSEA	ECVI
Pre-earthquake	GHQ	0.138	0.058	0.207
	Likert	0.136	0.057	0.205
	C-GHQ	0.098	0.048	0.167
Post-earthquake	GHQ	0.222	0.071	0.291
	Likert	0.225	0.072	0.294
	C-GHQ	0.196	0.066	0.266
Now	GHQ	0.180	0.067	0.249
	Likert	0.180	0.071	0.249
	C-GHQ	0.180	0.068	0.249

**Table 4:** Multiple regression analysis of factors that affected GHQ-12

	Total			Factor I			Factor II		
			
	Pre-	Post-	Now	Pre-	Post-	Now	Pre-	Post-	Now
Female gender		0.11	0.05		0.12	0.07	0.08	0.08	0.07
Age			0.06	0.06		0.11			
Place of residence when the earthquake occurred									
Nagaoka City						0.07			
Ojiya City			-0.14						
Mitsuke City							-0.06		
Kawaguchi Town	0.07			0.07	0.05	0.09			
Yamakoshi Village			0.10			0.14			
Employment									
Executive of business firm		-0.06	-0.05	-0.05	-0.06				
Office worker									0.05
Disemployment								-0.06	
Location when the earthquake occurred									
At home		0.05			0.06				
In public facilities							0.05		
No family members living together						0.04			
Lost income after earthquake						0.06			
Severity of house damage									
Severely damaged	0.08					0.10			
Almost completely destroyed				0.10		0.15			
Timing of contact with lifeguards									
1 to 3 hours after the earthquake		0.09			0.08				
3 to 6 hours		0.12			0.10				
6 to 12 hours								0.08	
more than 12 hours		0.16			0.15				
No contact							0.05	0.08	
Mild injury caused by the earthquake		0.06	0.10		0.06	0.10			
Mild sickness after the earthquake		0.07	0.09		0.08	0.09			
Adjusted R square	0.01	0.08	0.12	0.02	0.07	0.14	0.02	0.03	
